# The wounded territory: psychopolitic intervention strategy and resilience

**DOI:** 10.3402/ejpt.v4i0.22988

**Published:** 2013-10-30

## XIII ESTSS Conference: “Trauma and its clinical pathways: PTSD and beyond”, Bologna, June 2013 ORAL, JUNE 9 HALL LIZ

## Trauma e perdita

### Il territorio ferito: strategia psicopolitica d'intervento e resilienza   10:15–10:30

#### Ilaria Cinieri Istituto di Specializzazione in Psicoterapia Umanistico-Bioenergetico Psicoumanitas S.r.l.Taranto, Taranto, Puglia, Italy

La psicopolitica nasce nel 1976 dal pensiero e dal sentimento di Luigi De Marchi, e da egli stesso viene proposta come analisi dinamica dei grandi processi sociali. Utilizzando i suoi fondamenti abbiamo indagato la possibilità di *empowerment sociale* per riportare individuo e città ad uno stato di benessere, nella co-costruzione di *Social Holding*. Taranto è stata la città-teatro della nostra ricerca dal 2008 al 2012. Sviluppando ed attualizzando l'applicazione della tecnica analitica di De Marchi, è stata individuata una vera e propria strategia d'intervento che ha proposto - col *‘Social OUTing Project’* - nuovi modelli (ideali) di *Politica Ergonomica e Diritto al Benesserci* in risposta alla traumatogenicità eco-ambientale. La città era stata dichiarata in *dissesto finanziario* (2006) e piùrecentemente in stato di *disastro ambientale*, con un innalzamento della mortalità anche pediatrica (30% fino a 400%). È un osservatorio tristemente privilegiato per modalità e tempistica di attivazione ed organizzazione delle risorse complessive finalizzate alla resilienza. Il lavoro traduce l'idea della trasformazione e la volontà di condividere (Social Self Help Group), e proprio questi sembrano essere i principali bisogni accompagnati da vissuti di solitudine e abbandono registrati nella fase di ascolto. Attraverso vari contesti sociali è stato raccolto un campione rappresentativo dei vissuti individuo-generazionali, culturali e transculturali, e politico-amministrativi. Quali tecniche possono favorire l’ attivazione della resilienza ecosociale? Il metodo S.O.T., sperimentato nella mediazione cooperattiva, permette di intuirne il percorso di ristrutturazione dell'identità di chi abita un territorio ferito.

**Bibliografia**

De Marchi, L. (1984). *Scimmietta Ti Amo- da Neanderthal agli scenari atomici*. Edizioni Longanesi, Milano (IT).

Gordinier, J. (2008). *X saves the World*, Penguin. USA.

Lo Iacono, A., Milazzo, P. (2007). *La sala degli specchi: comunicazione e psicologia gruppale*. Franco Angeli, Milano (IT).

### The wounded territory: psychopolitic intervention strategy and resilience

The psychopolitical model, founded in 1976 by thoughts and feelings of Luigi De Marchi, was presented as a dynamic analysis of large social processes. Using its foundations we have checked the possibility of individual and social empowerment to bring the city to a state of well-being in building social holding.

Taranto was the home city of our research from 2008 to 2012. Developing and updating the application of the analytical technique of De Marchi, a real intervention strategy was found, which proposed — starting from the ‘Social Outing Project’—new ideal models of ergonomic policy and right to well-being in response to environmental traumas. The city was declared in financial difficulties (2006) and in a state of environmental disaster, and sees in the poisons of the ILVA steel factory the main causes of the increase of malignant diseases, cardiovascular, and respiratory mortality with a consequent increase of the pediatric one (30% up to 400%). Therefore, it is a sadly privileged observatory with regards to the modality and timing of activation and organization of resources aimed at the overall resilience. Through a methodology that continues his experiments within cooperative mediation, the work reflects the idea of transformation and the willingness to share (Social Self Help Group). These seem to be the main needs accompanied by feelings of loneliness and abandonment recorded in the listening phase. Through several layers and social contexts, a representative sample of individual experiences, generational, cultural, and cross-cultural as well as political-administrative was collected. Hence, what techniques will we have to reinterpret, learn, practice, and supervise, so that, they can be useful in enabling a resilient social/environmental/individual that facilitates the action in the real world, where even the generational proximity becomes a resource? The S.O.T. method used allows one to sense the process of restructuring the social identity and of those who are living in a wounded territory.

